# 3D-printed SAXS chamber for controlled *in situ* dialysis and optical characterization

**DOI:** 10.1107/S1600577522005136

**Published:** 2022-05-25

**Authors:** Tamara Ehm, Julian Philipp, Martin Barkey, Martina Ober, Achim Theo Brinkop, David Simml, Miriam von Westphalen, Bert Nickel, Roy Beck, Joachim O. Rädler

**Affiliations:** a School of Physics and Astronomy, Center for Physics and Chemistry of Living Systems, and Center for Nanoscience and Nanotechnology, Tel Aviv University, Ramat Aviv, Israel; b Faculty of Physics, Ludwig-Maximilians-Universität, Geschwister-Scholl-Platz 1, Munich, Germany

**Keywords:** cyclic olefin copolymer, 3D printing, *in situ* dialysis, in-house measurements, small-angle X-ray scattering

## Abstract

3D printing changes the scope of how samples can be mounted for small-angle X-ray scattering (SAXS). In this paper a 3D-printed X-ray chamber, which allows for *in situ* exchange of buffer and *in situ* optical transmission spectroscopy, is presented. The chamber is made of cyclic olefin copolymers (COC), including COC X-ray windows providing ultra-low SAXS background. The design integrates a membrane insert for *in situ* dialysis of the 100 µl sample volume against a reservoir, which enables measurements of the same sample under multiple conditions using an in-house X-ray setup equipped with a 17.4 keV molybdenum source. The design’s capabilities are demonstrated by measuring reversible structural changes in lipid and polymer systems as a function of salt concentration and pH. In the same chambers optical light transmission spectroscopy was carried out measuring the optical turbidity of the mesophases and local pH values using pH-responsive dyes. Microfluidic exchange and optical spectroscopy combined with *in situ* X-ray scattering enables vast applications for the study of responsive materials.

## Abstract 2

A 3D printed X-ray chamber which allows for *in situ* exchange of buffer and *in situ* optical transmission spectroscopy is presented.

## 1. Introduction

Small-angle X-ray scattering (SAXS) is a powerful technique for studying soft matter and biological materials at the nanometre scale (Hura *et al.*, 2013 ▸; Jacoby *et al.*, 2015 ▸; Kornreich *et al.*, 2015 ▸, 2016 ▸; Safinya *et al.*, 2015 ▸; Kikhney & Svergun, 2015 ▸). Apart from the high resolution, the key advantage of SAXS is its ability to resolve structures in solution under physiological conditions (Kler *et al.*, 2012 ▸; Mertens & Svergun, 2010 ▸; Chappa *et al.*, 2021 ▸; Brennich *et al.*, 2019 ▸). To this end fluid samples are traditionally filled in X-ray capillaries made of quartz glass. Samples are sealed and placed under equilibrated conditions in a high-flux X-ray beam at the synchrotron or in-house X-ray sources. Quartz capillaries suffer from curvature; therefore precise positioning is crucial for replicable results. Moreover, these studies require open systems, *e.g.* in time-resolved stop-flow and flow-through experiments, where repeated measurements are being conducted at different time delays in microfluidic channels (Abécassis *et al.*, 2007 ▸; Kihara, 1994 ▸; Krishnamoorthy *et al.*, 2019 ▸; Kirby *et al.*, 2013 ▸; Blanchet *et al.*, 2015 ▸). Structural changes in scattering can also be measured by rinsing in buffer within the microfluidic device and thus gradually changing the local concentration of, for example, salt or pH (Skou *et al.*, 2014 ▸; Junius *et al.*, 2020 ▸). A disadvantage of flow systems is that they cannot handle liquid crystalline or viscous mesophases, like lipid membrane systems or aggregated samples. They are useful, however, for studying the dynamics of aggregation, which are typically carried out in microfluidic devices with merging channels that allow continuous mixing (Toft *et al.*, 2008 ▸). In these devices it is key that a precisely positioned X-ray beam captures the temporal–spatial relationship of chemical and physical reactions (Denz *et al.*, 2018 ▸).

Here we design and test an *in situ* SAXS dialysis chamber which allows the exchange of buffer conditions in the X-ray sample holder. The design created from 3D-printed cyclic olefin co­polymer (COC) plastic fits to commercial dialysis membrane inserts and enables *in situ* SAXS experiments under varying buffer conditions, such as salinity, pH or osmolyte concentrations. We demonstrate modes of operation using responsive lipid and peptide amphiphile mesophases. Buffer exchange is controlled by diffusion on time-scales on the order of one hour allowing for repeated measurements of samples at different conditions on an in-house setup. In contrast to capillaries, our design has flat windows, facilitating positioning of the X-ray beam and reducing the sample-to-sample variation in sample thickness.

*In situ* SAXS dialysis allows for X-ray experiments on the same sample under continuously changing conditions, and thereby will decrease sample-to-sample variations in a series of SAXS measurements. In addition, the COC chamber design is comparatively easy to adapt and relatively cheap to produce. There are many examples where the relevant time-scale of structural rearrangement is slow and hence can also be followed using in-house X-ray sources, where radiation damage is minute, rather than synchrotron beamlines (Scattarella *et al.*, 2021 ▸; Jacoby *et al.*, 2015 ▸; Schmelzer Jr *et al.*, 2000 ▸). In these cases, in order to preserve precious samples, it is beneficial to only exchange buffer conditions via dialysis and to follow the structural dynamics *in situ* on the same sample.

In recent years several studies have presented microfluidic X-ray chambers made out of polymer materials, in particular the amorphous and optically transparent COC (Schwemmer *et al.*, 2016 ▸; Köster & Pfohl, 2012 ▸; Silva *et al.*, 2015 ▸; Ghazal *et al.*, 2016 ▸; Denz *et al.*, 2018 ▸). This approach, using an X-ray transparent chip, allows for *in situ* sample mixing during SAXS measurements or *in situ* dilution of, for example, proteins with a variety of buffers. COC materials are also frequently used in crystallography screening by using 96 well plates made out of COC (Lee *et al.*, 2013 ▸; Joseph *et al.*, 2011 ▸). Traditionally, micro-fluidic X-ray chambers use the well established window material Kapton, which has rather low optical transparency and needs adhesives for attachment to the chamber. COC devices have the advantage that they can be manufactured using 3D printing. In addition, foils from the same material can be used as X-ray window material.

Annealing the foils to printed structures provides a simple, precise and reproducible production of sample chambers. Most importantly, COCs are well suited for X-ray applications and optical measurements due to their high optical transparency and low background scattering at relevant photon energies as compared with PDMS and SiO (Guha *et al.*, 2012 ▸). Previous studies (Denz *et al.*, 2018 ▸) have benchmarked COC versus Kapton windows with gold colloid measurements at two different synchrotron beamlines. The background-subtracted scattering data of the COC device and of the Kapton device agreed very well. So far, no COC chamber has been reported that allows for buffer exchange.

## 2. Chamber design

**Figure 1:**
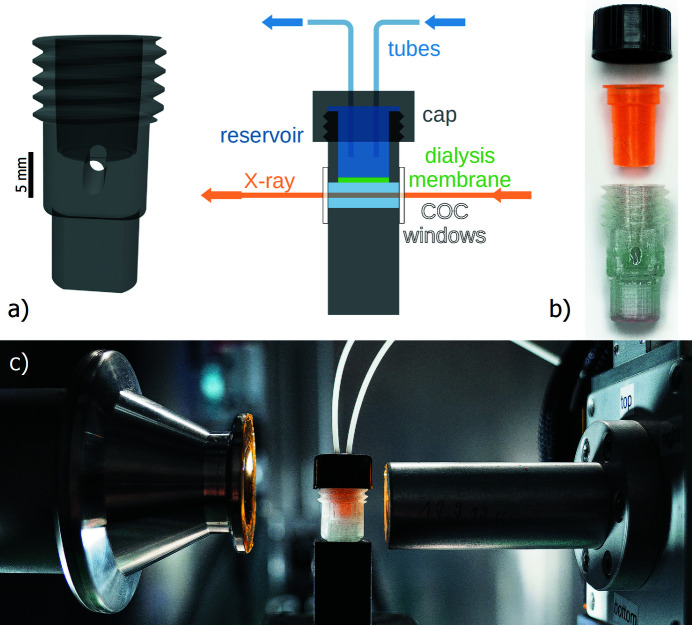
Chamber design principle and implementation. (*a*) Schematic drawing of the COC chamber. (*b*) Sample corpus is 3D-printed out of COC to enclose the coloured dialysis tube. Two COC X-ray windows allow for measurement of both condensed and diluted phases. (*c*) Sample setup for *in situ* measurements and dialysis using syringe pump systems.

Our key design idea was to create a SAXS-compatible sample chamber with *in situ* dialysis capabilities. In order to avoid impractical handling with loose dialysis membranes, we used commercial dialysis inserts for Eppendorf cups (Slide-A-Lyzer MINI 0.1 ml, ThermoFisher). The SAXS chamber was built to create a small sample chamber in close proximity to the dialysis membrane. These dialysis inserts provide a 500 µL liquid reservoir, which can be extended via external syringe pumps. The dilution with just the 500 µL liquid reservoir is sufficient for small pH/salt changes; we recommend using the syringe pumps for more significant steps. The dialysis inserts have a standardized size, are easily mountable, and come in various different pore sizes. For our purposes we used a molecular weight cutoff of 3.5 kDa. The surrounding sample chamber is first 3D printed with COC using an Ultimaker 3 and then sealed on both sides with transparent windows made of COC foil (Fig. 1 ▸). Because both the chamber and the windows are made of the same polymers, they can be annealed by simply heating both for a few seconds on a hotplate at 160°C. For our chambers we use 2.85 mm-thick Creamelt^TM^ COC filament (Herz GmbH) and 50 µm-thick TOPAS^TM^ 8007F-04 COC foil (Microfluidic ChipShop).

The chamber can be filled from the top before closing it off by mounting the dialysis insert, which itself can be closed by a simple cap to avoid evaporation of the reservoir. The sample volume below the dialysis membrane is about 100 µl and its optical path length is optimized for a 17.4 keV molybdenum source. If necessary, the reservoir can be continuously exchanged via syringe pumps and tubes through the cap to increase the reservoir volume. For time-resolved experiments, the chamber can be placed in the X-ray beam path where syringe pumps can exchange the reservoir buffer *in situ* (Fig. 1 ▸).

**Figure 2:**
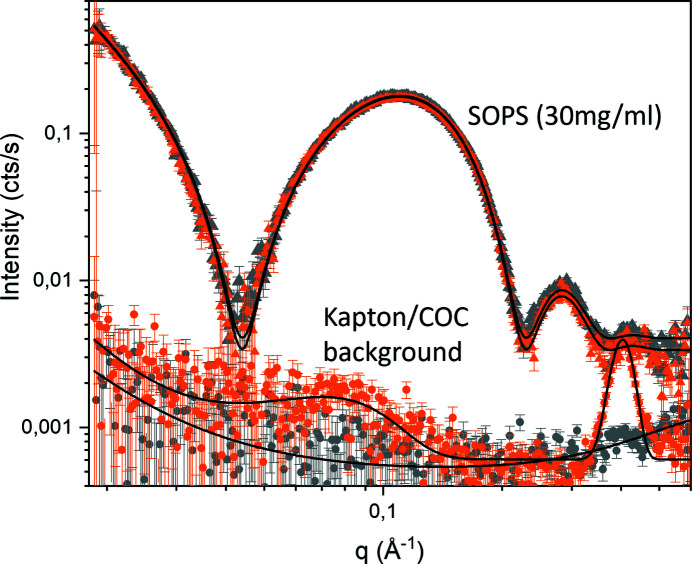
In-house measurements with Kapton and COC windows. Triangles: SAXS signal with error bars (orange/grey: Kapton/COC windows) of SOPS unilamellar vesicles with a diameter of 100 nm (30 mg ml^−1^). Solid lines correspond to the best fit for flat symmetrical bilayers (see supporting information for model and detailed fit parameters). Circles: SAXS signal with error bars of the empty dialysis chamber with 50 µm-thick Kapton (orange) and 100 µm-thick COC windows (grey). Solid lines correspond to the best fit for a power law with one and two Gaussians. The samples were measured with an exposure time of 1 min per frame and measured for 6 h in total.

We first characterized the signal-to-noise ratio of X-ray scattering in our dialysis SAXS chamber for both COC and Kapton windows, using unilamellar vesicles made of SOPS at 30 mg ml^−1^ concentration (Fig. 2 ▸). The scattering signal is in agreement for both of the X-ray window materials; however, background measurements with the empty 3D-printed chamber showed that Kapton windows have a slightly stronger background signal than COC. This is most visible in the *q*-regimes where Kapton is known to exhibit scattering, *i.e.* a broad peak around *q* = 0.09 Å^−1^ as well as in the wide-angle X-ray scattering (WAXS) region with scattering at *q* = 0.4 Å^−1^ depending of the type of Kapton.

## 3. Buffer exchange kinetics

**Figure 3:**
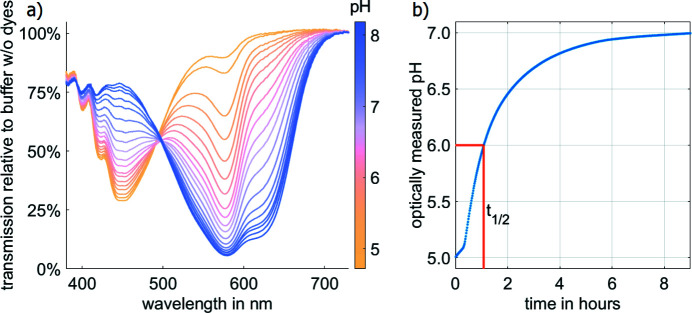
Time-scale of *in situ* pH change. (*a*) pH-dependent transmission spectra of bromothymol blue and chlorophynol red mixed at a molar ratio of 2:1. (*b*) Optical measurements of the pH inside the sample chamber during dialysis results in an approximate exchange half-life time of *t*_1/2_ = 1 h.

Buffer is exchanged between the reservoir and the sample volume via diffusive transport across the built-in dialysis membrane. For our design this reversible dialysis occurs over a time-scale of a few hours. Our COC chamber allows for both optical and X-ray measurements of the sample in the same environment. We used this feature to follow the dialysis optically. Mixed at a ratio of 1:2, we used the pH indicators chlorophenol red and bromothymol blue, which change colour from yellow to pink to blue for a pH range of 5 to 8. By preparing several samples within this range and measuring their transmission spectra in the chamber, we were able to calculate the spectra depending on the pH and vice versa [Fig. 3 ▸(*a*)]. This allows for optical pH measurements without disturbing the sample during dialysis. Fig. 3 ▸(*b*) shows the time evolution of pH inside our chamber during dialysis, which gives a good estimate of the diffusion times (*t*_1/2_ = 1 h). The result is in agreement with the calculated diffusion times (*t* = 1.25 h) via *t* = 〈*x*〉^2^/*D*, where *x* is the vertical diffusion path along the chamber (*x* ≃ 3 mm) and *D* is the typical diffusion coefficient for buffer salts (*D* ≃ 10^−5^ cm^2^ s^−1^).

**Figure 4:**
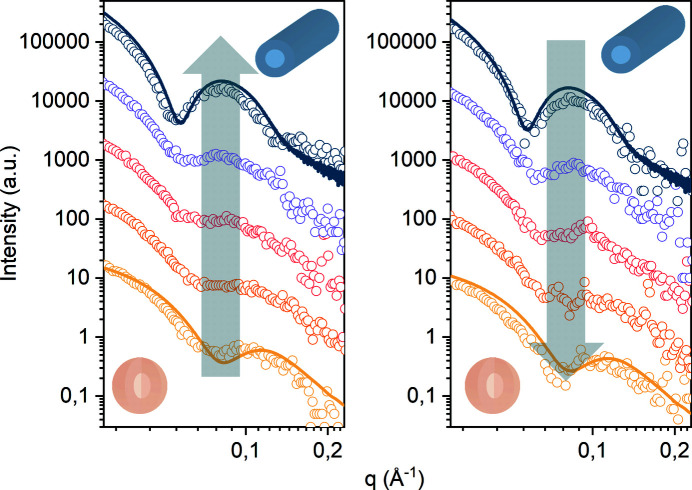
pH-dependent phase transition in an amphiphilic peptide mesophase. Left: time-resolved in-house SAXS measurement (background corrected) of *in situ* dialysis with 3D-printed dialysis chamber from pH 7.5 to pH 4.7 of IDPAs shows a transition from micellar rods to spherical micelles in less than 6 h. Scattering curves are snapshots at 0, 140, 160, 180 and 220 min with 20 min exposure time each. Solid lines are convoluted reference scattering curves from high-resolution SAXS beamlines (data were taken with an automated sample robot at DESY, Hamburg, Germany). Right: pH-dialysis back from pH 4.7 to pH 7.5 shows the reversibility of the transition in the dialysis chamber. Scattering curves are snapshots at 0, 260, 240, 340 and 520 min with 20 min exposure time each.

To show reversibility, we used a pH-responsive system of intrinsically disordered peptide amphiphiles [IDPAs (Jacoby *et al.*, 2021 ▸)]. Specific amino acids in the used sequence can get protonated and become uncharged with decreasing pH. At pH 4.7 the IDPAs self-assemble into rod-like micelles. Upon increasing the pH to pH 7.5, the IDPAs transform into spherical micelles with a change in form factor scattering. We were able to monitor this transition from micellar rods to spherical rods and vice versa (pH 4.7 to pH 7.5) in an in-house SAXS source (see supporting information). As the diffusion time-scales for the transition points are larger than the time resolution of the in-house SAXS device, these experiments do not require a high-resolution synchrotron beam. This makes measurement cycles easily applicable at home sources. We monitored the transition with time-resolved SAXS measurements (Fig. 4 ▸). The SAXS data did not show any changes after 6 h.

## 4. Experimental results

**Figure 5:**
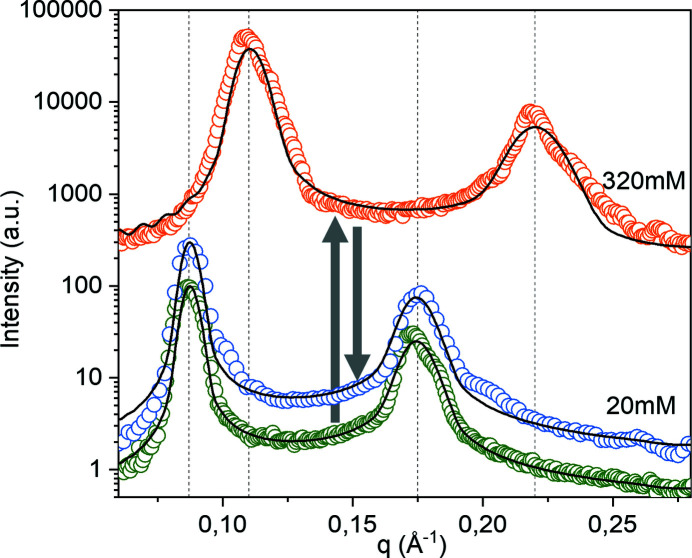
Reversible exchange of ionic buffer conditions. In-house SAXS signal of a charged lamellar phase (Soy PC doped with 5 wt% DOTAP) exhibits the shrinking and subsequent swelling of the intermembrane distance in response to increasing and decreasing salt concentrations. Black lines indicate fits of the data using the modified Caillé theory (MCT) combined with a Gaussian electron density representation, as proposed by Pabst *et al.* (2000 ▸). The sample was equilibrated for seven days.

By conducting measurements with our 3D-printed dialysis chamber, we demonstrated its usability in pH exchange. In principle, the chamber can be used for any buffer exchange where the diffusion molecules have a lower molecular weight than the cut-off of the dialysis membrane, *e.g.* the exchange of salt often used in monitoring and controlling electrostatic interactions in biological SAXS experiments. With our 3D-printed COC chamber, salt concentration series can now be performed in a single sample. An exemplary measurement is shown in Fig. 5 ▸. Here, l-α-phosphatidylcholin (Soy PC) doped with 5 wt% of 1,2-dioleoyl-3-trimethylammoniumpropan (DOTAP) was dialysed from 20 m*M* NaCl to 320 m*M* NaCl. The decrease in electrostatic repulsion due to charge screening resulted in shrinkage of the intermembrane distance (300 m*M*: 6.0 nm; 20 m*M*: 5.7 nm). The dialysis was reversed by exchanging the reservoir volume until the original salt concentration was restored. During this process, the lamellar repeat distance exhibited subsequent swelling.

**Figure 6:**
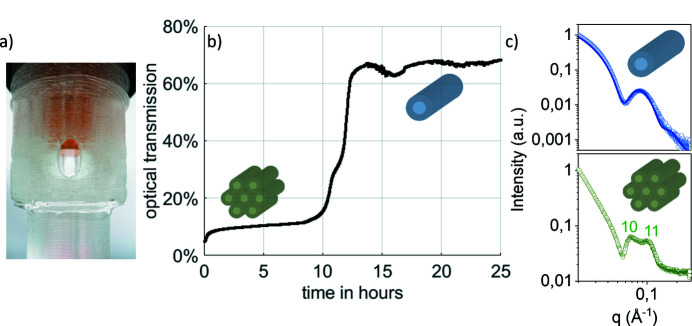
(*a*) Photograph of the X-ray chamber showing the optical transparent X-ray window and the dialysis insert (orange). (*b*) Optical turbidity across the X-ray chamber showing the structural transition from a condensed hexagonal to a dispersed cylindrical micelle phase. (*c*) In-house SAXS signal of hexagonal-packed (green) and monodispersed micellar rods (blue) in solution. Data were taken with 20 min exposure time each. The solid line corresponds to the spherical-core shell form factor (blue). Miller indices show first and second peaks for 2D hexagonal lattice.

Additionally, the design allows for absorbance measurements of (non-) turbid samples. Thus time-scales of phase transitions that exhibit a change in turbidity can be screened. Here, we examined the turbidity of the system of IDPAs presented in the last section. They undergo a phase transition from micellar, monodisperse, non-turbid rods at intermediate pH into hexagonal packed rods at the isoelectric point (pH 4). The hexagonal phase has higher absorption rates, as these mesophases are in the size range of the wavelength of the applied light (Fig. 6 ▸).

## 5. Conclusion

In this paper, we designed and characterized a 3D-printed SAXS dialysis chamber made out of COC, and showed that the device is well suited for in-house X-ray measurements. The chamber allowed for *in situ* buffer exchange within a half-life time of about 1 h as determined using pH-sensitive dyes. We showed that pH-sensitive amphiphilic peptide mesophases underwent structural changes in a reversible fashion when pH was cycled from high to low pH and back to high pH. Likewise, continuous change of ionic strength exhibited predicted shrinking and swelling of the spacing in charged lamellar lipid phases. Optical spectroscopy was carried out on the same samples within the X-ray chamber thanks to the optical transparency of the COC windows. The fabrication of larger numbers of X-ray chambers using 3D printing is simple and reliable. In contrast to regular capillaries, our 3D-printed X-ray chamber is connected to a reservoir via a dialysis membrane and, therefore, allows for multiple measurements of the same sample under different buffer conditions. This approach saves sample material that is sometimes limited or costly. Most importantly using *in situ* dialysis renders differential SAXS measurements highly reliable by avoiding sample-to-sample variations, which are typically caused by impreciseness in sample preparation or variations between sample holders. In our design the chamber dimensions were optimized for a molybdenum 17.9 keV X-ray source with attenuation length in water of about 1 cm (Bruetzel *et al.*, 2016 ▸). In this case the integration of a commercial dialysis insert was easily achievable. In principle a smaller sample volume is desirable since, firstly, in-house SAXS sources with copper anodes could be used, and, secondly, diffusion-limited buffer exchange would accelerate. However, a smaller sample volume requires smaller dialysis inserts and comes with the difficulties of laminating the chamber windows on an even smaller 3D-frame. Future chamber designs might overcome these problems. Alternatively, faster exchange kinetics in the existing design could be achieved by active mixing, *e.g.* using magnetic micro-stirrers. The latter might also be required if dense samples exhibit strongly reduced diffusion times. In summary, 3D-printed sample holders are promising for *in situ* X-ray scattering providing microenvironments that enable the integration of continuous buffer exchange and inspection by optical spectroscopy.

## 6. Data availability statement

The cura profile to print is openly available from https://3dprint.nih.gov/discover/3dpx-016474, as version 01. Updates will be uploaded.

## Supplementary Material

Supporting information file. DOI: 10.1107/S1600577522005136/ju5042sup1.pdf
