# Supplementary material for: 3D-printed SAXS chamber for controlled in situ dialysis and optical characterization
Source: J Synchrotron Radiat. 2022 May 25;29(Pt 4):1014–9. doi: 10.1107/S1600577522005136 (PMC9255564; doi:10.1107/S1600577522005136)
Supplement: Supplementary file 1 [file s-29-01014-sup1.pdf]

JOURNAL OF SYNCHROTRON RADIATION

Volume 29 (2022)

Supporting information for article:

3D printed SAXS chamber for controlled *in-situ* dialysis and optical characterization

Tamara Ehm, Julian Philipp, Martin Barkey, Martina Ober, Achim Theo Brinkop, David Simml, Miriam von Westphalen, Bert Nickel, Roy Beck and Joachim O. Rädler

S.1. Supporting Information

The following parameters were used for printing the COC chamber:

Printer	Ultimaker 3
Material	COC 2.85
Nozzle Size	0.4 mm
Slicing	Cura 4.6.1
Layer Height	0.15 mm
Wall Thickness	1.3 mm
Top/Bottom Thickness	1.2 mm
Horizontal Expansion	-0.05 mm
Infill Density	20 %
Printing Temperature	245 °C
Build Plate Temperature	80 °C
Printing Speed	60 mm/s
Generate Support	None
Build Plate Adhesion Type	Brim

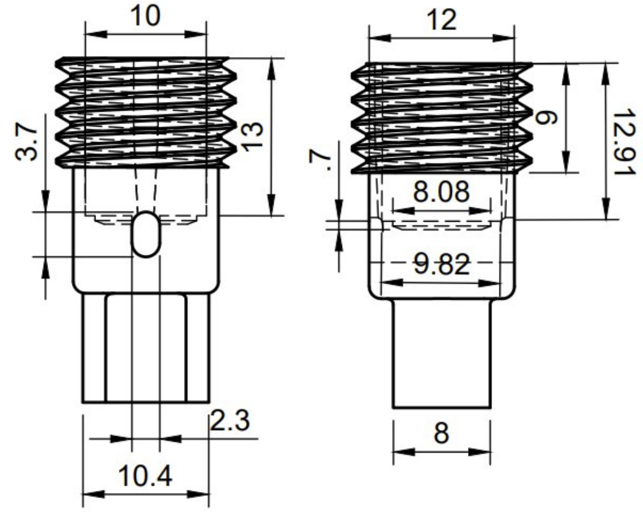

Fig. S.1. **Technical drawing of 3D printed chamber.** Front and side view of chamber, dimensions are given in mm.

S.1.1. Experimental section about SAXS experiments

Our in-house SAXS system consists of a Mo GeniX3D microfocus X-ray tube (Xenocs SA, Sassenage, France) combined with FOX2D single reflection optics delivering a monochromatic and highly stable beam with an X-ray energy of $17.4keV$. The

flux is typically around 2.5×106 photons/s at the sample stage. For collimation the beam enters an $82cm$ long, fully evacuated collimation path closed by a 25 µm thick Kapton foil at the end. Collimation is achieved by integrating two partially motorized scatterless aperture slits (Xenocs SA, Sassenage, France), one upstream right at the mirror and the second at the tube exit.The sample stage is positioned 5 cm in front of the collimation path exit. Sample to detector distance is 1m. For Fig. 2 samples were measured with an exposure time of one minute per frame for 6 hours in total, for Fig. 4 and 5 with a total exposure time of 20 min in one frame. SAXS measurements for synchrotron data for Fig. 6 were done at beamline B21 (Cowieson *et al.*, 2020) and I22 (Smith *et al.*, 2020), high-throughput small-angle X-ray scattering at Diamond Light Source. Here the beamsize is 75 μm and a 4M Eiger detector is used. For sample environment we used the sample robot at B21 for fluid samples (rod, blue curve in Fig. 6) and quartz capillaries at I22 for sedimented samples (hexagonal phase, green curve in Fig. 6).

S.1.2. Small angle X-ray scattering (SAXS) analysis of SOPS LUVs data

All bilayer parameters are obtained from model fits of the background corrected total scattering intensity $I(q)$ to an electron density profile $\Delta\rho(z)$ composed of three Gaussians:

$$\Delta\rho(z) = \Delta\rho_H \ \exp\left[-\frac{(z-z_H)^2}{2\sigma_H^2}\right] + \Delta\rho_{CH} \ \exp\left[-\frac{z^2}{2\sigma_{CH}^2}\right] + \Delta\rho_H \ \exp\left[-\frac{(z+z_H)^2}{2\sigma_H^2}\right]$$

Here, $\Delta\rho_H$ is the scattering length contrast of the lipid head groups compared to water, i.e. $\Delta\rho(z) = \Delta\rho_H - \ \Delta\rho_w$. z_H is the spatial peak offset of the head centers in respect to the center of the bilayer, and σ_H is the corresponding variance of the Gaussian functions. $\Delta\rho_{CH}$ is the scattering length contrast of the lipid chains and σ_{CH} the variance of the Gaussian function describing the chain region. Model fitting was achieved by running the software-internal Levenberg-Marquardt algorithm using

the software package SasView (http://www.sasview.org/).

Data	$z_H [\AA]$	$D\rho_{C\|H} [a.u.]$	$\sigma_{CH} [\AA]$	$\Delta\rho_H [a.u.]$	$\sigma_H [\AA]$
COC	21.1	-0.002	8.6	0.004	4.3
Kapton	20.9	-0.003	9.6	0.005	4.5

Table S.1. *Parameters obtained from least-squares fitting of SAXS data of 100 nm extruded SOPS vesicles to a symmetrical flat bilayer model.*

References

Cowieson, N. P., Edwards-Gayle, C. J., Inoue, K., Khunti, N. S., Doutch, J., Williams, E., Daniels, S., Preece, G., Krumpa, N. A., Sutter, J. P. *et al.* (2020). *Journal of Synchrotron Radiation*, **27**(5), 1438–1446.

Smith, A., Alcock, S., Davidson, L., Emmins, J., Hiller Bardsley, J., Holloway, P., Malfois, M., Marshall, A., Pizzey, C., Rogers, S. *et al.* (2020). *Journal of synchrotron radiation*, **28**(3), 939–947.
